# Factors Affecting the Accuracy of Controlled Attenuation Parameter (CAP) in Assessing Hepatic Steatosis in Patients with Chronic Liver Disease

**DOI:** 10.1371/journal.pone.0098689

**Published:** 2014-06-05

**Authors:** Kyu Sik Jung, Beom Kyung Kim, Seung Up Kim, Young Eun Chon, Kyung Hyun Cheon, Sung Bae Kim, Sang Hoon Lee, Sung Soo Ahn, Jun Yong Park, Do Young Kim, Sang Hoon Ahn, Young Nyun Park, Kwang-Hyub Han

**Affiliations:** 1 Department of Internal Medicine, Yonsei University College of Medicine, Seoul, Korea; 2 Institute of Gastroenterology, Yonsei University College of Medicine, Seoul, Korea; 3 Department of Pathology, Yonsei University College of Medicine, Seoul, Korea; 4 Liver Cirrhosis Clinical Research Center, Seoul, Korea; 5 Brain Korea 21 Project of Medical Science, Seoul, Korea; The University of Hong Kong, Hong Kong

## Abstract

**Background & Aims:**

Controlled attenuation parameter (CAP) can measure hepatic steatosis. However, factors affecting its accuracy have not been described yet. This study investigated predictors of discordance between liver biopsy (LB) and CAP.

**Methods:**

A total of 161 consecutive patients with chronic liver disease who underwent LB and CAP were enrolled prospectively. Histological steatosis was graded as S0 (<5%), S1 (5–33%), S2 (34–66%), and S3 (>66% of hepatocytes). Cutoff CAP values were calculated from our cohort (250, 301, and 325 dB/m for ≥S1, ≥S2, and S3). Discordance was defined as a discrepancy of at least two steatosis stages between LB and CAP.

**Results:**

The median age (102 males and 59 females) was 49 years. Repartition of histological steatosis was as follows; S0 26.1% (n = 42), S1 49.7% (n = 80), S2 20.5% (n = 33), and S3 3.7% (n = 6). In multivariate linear regression analysis, CAP value was independently associated with steatosis grade along with body mass index (BMI) and interquartile range/median of CAP value (IQR/M_CAP_) (all P<0.05). Discordance was identified in 13 (8.1%) patients. In multivariate analysis, histological S3 (odd ratio [OR], 9.573; 95% confidence interval [CI], 1.207–75.931; *P* = 0.033) and CAP value (OR, 1.020; 95% CI, 1.006–1.034; *P* = 0.006) were significantly associated with discordance, when adjusting for BMI, IQR/M_CAP_, and necroinflammation, reflected by histological activity or ALT level.

**Conclusions:**

Patients with high grade steatosis or high CAP values have a higher risk of discordance between LB and CAP. Further studies are needed to improve the accuracy of CAP interpretation, especially in patients with higher CAP values.

## Introduction

Currently, the clinical implications of hepatic steatosis are gaining more attention not only in Western countries, but also in Asian countries with a westernized lifestyle such as Japan, China, and Korea. [1], [2] Indeed, the incidence of non-alcoholic fatty liver disease (NAFLD), the most common condition of steatosis, is increasing worldwide and it is now the most common cause of abnormal liver function tests and chronic liver disease (CLD) in both developed and developing countries. [3].

Severe forms of NAFLD can cause serious liver-related complications such as liver failure and hepatocellular carcinoma. [4], [5] Furthermore, fatty burden can negatively influence the prognosis of patients with CLD, as reported by recent studies revealing that coexistent steatosis in chronic hepatitis C is associated with fibrosis progression and decreased treatment response, and that steatosis may lead to a poor postoperative outcome such as a graft failure after liver transplantation or high mortality after hepatectomy. [6]–[9] Moreover, these studies also suggested that even low burden of hepatic steatosis could affect treatment outcome or prognosis. Thus, an accurate diagnosis and objective estimation of hepatic steatosis is important for clinical decision-making and estimating the prognosis.

To date, the gold standard for diagnosing and assessing the severity of hepatic steatosis has been liver biopsy (LB). [10] However, LB is an invasive and costly procedure with potential limitations such as sampling error and unsatisfactory reproducibility. [11], [12] Moreover, LB is difficult to repeat and it allows only semiquantitative grading of steatosis. Although several non-invasive methods such as ultrasonography, computed tomography (CT), and magnetic resonance imaging (MRI) have been investigated for this purpose, their clinical use is limited by high cost, restrictive availability, operator dependence, and poor sensitivity. [13], [14].

Recently, interest has shifted towards controlled attenuation parameter (CAP), which is based on the properties of ultrasonic signals acquired by transient elastography (TE). [15] Previous studies have demonstrated that CAP can be performed rapidly, and painlessly with high patient acceptance and that it can accurately grade the severity of steatosis in patients with CLDs. [15]–[18] However, in contrast to a situation that several confounding factors which determines the accuracy of liver stiffness (LS) values such as interquartile range/median value (IQR/M) or necroinflammatory activity have been identified, factors that affect the accuracy of CAP in assessing hepatic steatosis have not yet been identified. [19]–[22] Here, this study investigated factors which can influence the diagnostic accuracy of CAP for estimating the severity of hepatic steatosis.

## Patients and Methods

### Patients

Between November 2011 and August 2013, patients with CLD of any etiology who were scheduled to undergo LB and CAP were recruited for this prospective study at Severance Hospital, Yonsei University College of Medicine, Seoul, Korea. The indications of LB were (i) assessing the degree of inflammatory activity and the extent of liver fibrosis in patients with viral hepatitis B and C, and (ii) establish the cause of the liver disease in patients without viral hepatitis. Exclusion criteria were as follows; (1) TE measurement failure (no valid shot); (2) unreliable LS measurement; (3) non-interpretable biopsies including insufficient specimen size <10 mm in length. Written informed consent was obtained from all patients before enrollment. The study protocol was consistent with the ethical guidelines of the 1975 Declaration of Helsinki. Written informed consent was obtained from each participant. This study was approved by the Ethics Committee/independent institutional review board of Severance Hospital, Yonsei University College of Medicine, Seoul, Korea. The cohort of this study included subjects recruited in our previous study [23] and we enrolled additional subjects in the currnet study.

### Measurement of Liver Stiffness and Controlled Attenuation Parameter

All patients underwent TE using the Fibroscan M probe on the same day as LB after fasting for at least 8 hour. [24] TE was performed on the right lobe of the liver through the intercostal spaces with the patient lying in the dorsal decubitus position with the right arm in maximal abduction. Only one experienced technician blind to the patients’ clinical data, was allowed to perform TE. The principles of CAP measurement have been described previously. [15] Briefly, the CAP measures ultrasonic attenuations at 3.5 MHz using signals acquired by TE.

TE results were expressed as kilopascals (kPa) for LS and dB/m for CAP. The interquartile range (IQR) was defined as an index of the intrinsic variability of LS and CAP values corresponding to the interval of LS and CAP results containing 50% of the valid measurements between the 25^th^ and 75^th^ percentiles. The median value of the successful measurements was selected as representative of LS and CAP values for a given patient. As an indicator of variability, the ratio of the IQR of LS and CAP values to the median (IQR/M and IQR/M_CAP_, respectively) was calculated.

At the same time, hepatic steatosis was assessed using CAP value, only when LS measurement was valid for the same signals, ensuring that the liver ultrasonic attenuation was obtained simultaneously from the same volume of liver parenchyma as LS measurement. In this study, only TE measurement with at least 10 valid shots, and a success rate of at least 60% were considered reliable and used for statistical analysis.

### Clinical Data

Before TE examination, demographics, liver disease etiology and anthropometric measurements were obtained. Biochemical parameters including aspartate aminotransferase (AST), alanine aminotransferase (ALT), platelet count, serum fasting glucose, total cholesterol, and triglycerides were measured on the same day as the LB.

### Liver Biopsy

All patients underwent ultrasound-guided percutaneous LB. The LB specimens were fixed in formalin and embedded in paraffin. Then, 4-µm-thick sections were subjected to hematoxylin-eosin and Masson’s trichrome staining. All liver tissue samples were evaluated by an experienced hepatopathologist (YN Park) who had no access to the clinical data on the study population. Liver fibrosis stage and necroinflammation were evaluated using the Metavir or Brunt scoring system, according to the liver disease etiology. [25], [26] Steatosis was assessed as the percentage of hepatocytes containing lipid droplets and categorized according to the NAFLD Activity Score (S0, <5%, S1, 5–33%; S2, 34–66%; and S3, >66%). [27].

### Statistical Analyses

Data are expressed as means ± SD, median (range), or n (%), as appropriate. ‘***Discordance***’ was defined as a difference of at least two steatosis stages between LB and CAP. Correlations between variable were described using Spearman correlation coefficients (ρ). Comparisons between patients with discordance and those without were made using the Student *t*-tests or Mann-Whitney test for continuous variables and chi-squared or Fisher’s exact test for categorical variables. Cutoff CAP values to determine discordance were calculated from our cohort, which maximized the sum of the sensitivity (Se) and specificity (Sp) (Youden index). Positive and negative predictive values (PPV and NPV) were also computed. Areas under the receiver operating characteristic curves (AUROC) were computed with 95% confidence intervals (CI). Univariate and subsequent multivariate binary logistic regression analyses were performed to identify independent factors of discordance between LB and CAP. Odd ratios (ORs) and corresponding 95% CI were also indicated. A *P* value <0.05 on a two-tailed test was considered significant. Data analyses were performed using the SAS program (ver. 9.1; SAS Inc., Cary, NC, USA).

## Results

### Baseline Characteristics

**Figure 1:**
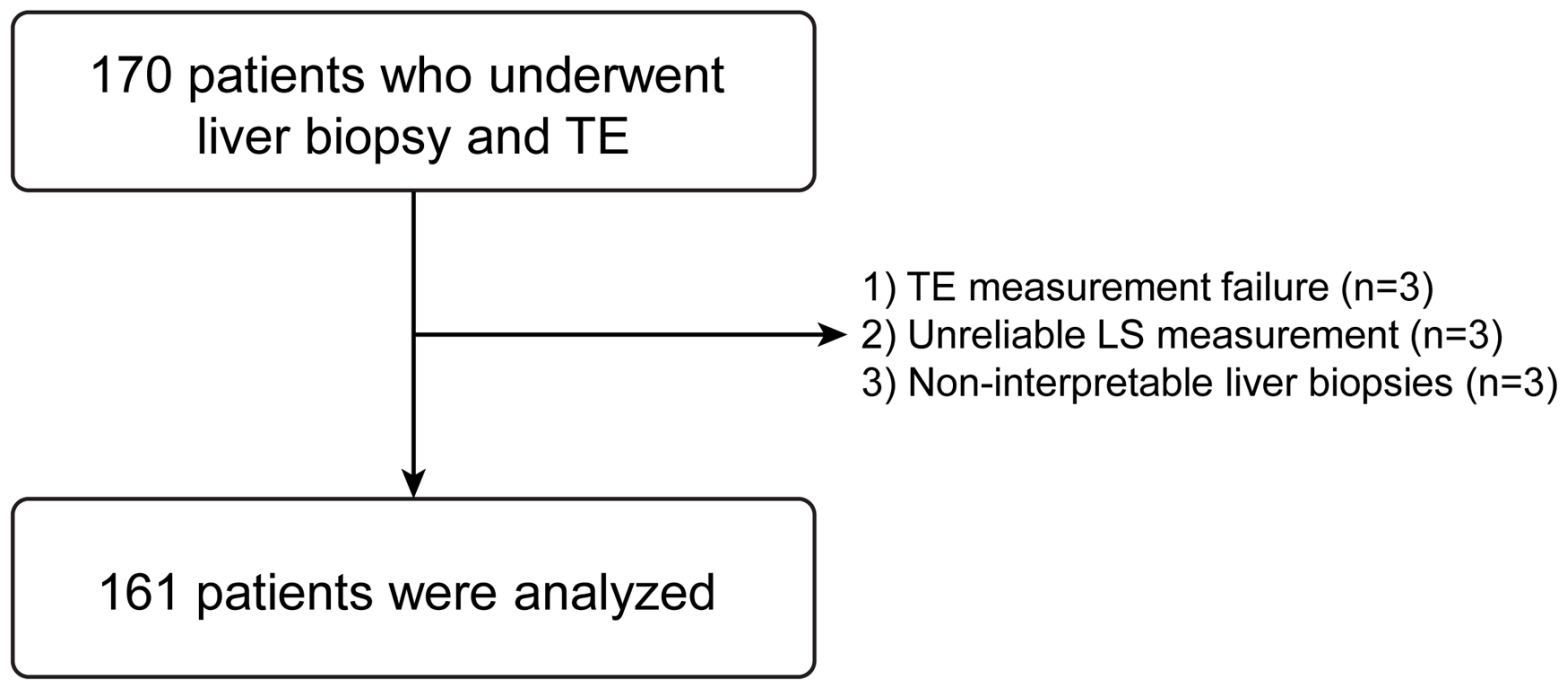
Recruitment algorithm. A total of 170 patients with CLD were consecutively enrolled. However 9 patients were excluded due to TE measurement failure (n = 3), unreliable LS measurement (n = 3), non-interpretable liver biopsies (n = 3), leaving 161 patients to be included in the statistical analysis. CLD, chronic liver disease; TE, transient elastography; LS, liver stiffness.

During the study period, 170 patients with CLD (135 patients who were recruited in our previous study [23] and additional 35 patients) were enrolled. However, after excluding 9 patients based on our exclusion criteria, 161 patients were selected for statistical analysis (**Fig. 1**). The baseline characteristics of the study are summarized in **Table 1**. The majority were male (n = 102, 63.4%) and the median age was 49 years. The median body mass index (BMI) was 24.4 kg/m2 and 28 (17.4%) patients had diabetes mellitus. The main etiologies of CLD were NAFLD (n = 72, 44.7%), followed by chronic viral hepatitis including chronic hepatitis B (n = 49, 30.4%) and C (n = 28, 17.4%), autoimmune hepatitis (n = 8, 5.0%) and primary biliary cirrhosis (n = 4, 2.5%). The median LS and CAP values were 8.1 (range, 2.9–75.0) kPa and 255 (range, 149–400) dB/m, respectively. The median IQR/M and IQR/MCAP were 0.12 (range, 0.01–0.35) and 0.12 (range, 0.02–0.33), respectively and IQR/M was higher than 0.3 in only one patient.

**Table 1:** Baseline characteristics (n = 161).

Variables	Values
**Demographic variables**	
Age (years)	49 (18–81)
Male gender	102 (63.4)
Body mass index (kg/m^2^)	24.4 (14.3–34.3)
Diabetes mellitus	28 (17.4)
Etiology, NAFLD/HBV/HCV/Others	72 (44.7)/49 (30.4)/28 (17.4)/12 (7.5)
**Biochemical parameters**	
Aspartate aminotransferase (IU/L)	45.2±36.2
Alanine aminotransferase (IU/L)	49.8±43.8
Serum albumin (g/dL)	4.1±0.5
Fasting glucose (mg/mL)	109.6±35.2
Total cholesterol (mg/mL)	172.2±40.5
Triglycerides (mg/mL)	128.5±60.3
**Liver biopsy**	
Fibrosis stage	
F0–1/F2/F3/F4	65 (40.4)/44 (27.3)/22 (13.7)/30 (18.6)
Activity grade	
A0/A1/A2/A3	23 (14.3)/27 (16.8)/74 (46.0)/37 (23.0)
Steatosis	
S0/S1/S2/S3	42 (26.1)/80 (49.7)/33 (20.5)/6 (3.7)
Biopsy length (cm)	18.5 (16.1–24.2)
**Liver stiffness measurement**	
Liver stiffness value (kPa)	8.1 (2.9–75.0)
Interquartile range (kPa)	1.0 (0.1–14.0)
IQR/M	0.12 (0.01∼0.35)
**Controlled attenuation parameter**	
Controlled attenuation parameter value (dB/m)	255 (149–400)
Interquartile range (dB/m)	29.0 (6–76)
IQR/M_CAP_	0.12 (0.02–0.33)

Variables are expressed as median (range) or n (%). NAFLD, non-alcoholic fatty liver disease; HBV, hepatitis B; HCV, hepatitis C; kPa, kilopascal; IQR/M, interquartile range/median liver stiffness value; CAP, controlled attenuation parameter; IQR/M_CAP_, interquartile range/median of CAP value.

### Liver Histology and Corresponding CAP Value

The median length of the LB specimens was 18.5 (range, 16.1–24.2) mm. The histological steatosis grade was S0 in 42 (26.1%) patients, S1 in 80 (49.7%), S2 in 33 (20.5%), and S3 in 6 (3.7%), respectively. The median CAP values for patients with S0, S1, S2, and S3 steatosis on LB were 217 (range, 149–288), 258 (range, 150–345), 331 (range, 234–400), and 326 (range, 230–347) dB/m, respectively. The median and range of CAP values according to fibrosis stage and activity grade are listed in **Table S1 in File S1**. CAP values were not significantly different between each fibrosis stage and activity grade (all *P*>0.05).

The cutoff CAP values determining ≥S1, ≥S2, and S3 were calculated as 250 dB/m (AUROC, 0.863 [95% CI, 0.807–0.920]; Se, 68.9%; Sp, 92.9%; PPV, 96.5%; NPV, 51.3%), 301 dB/m (AUROC, 0.898 [95% CI, 0.841–0.954]; Se, 82.1%; Sp, 87.7%; PPV, 68.1%; NPV, 93.9%), and 325 dB/m (AUROC, 0.738 [95% CI, 0.562–0.914]; Se, 50.3%; Sp, 81.3%; PPV, 9.4%; NPV, 97.7%), respectively.

### Correlation between CAP and Clinicopathological Variables

In univariate linear regression, CAP value was significantly associated with BMI (*P*<0.001), ALT (*P* = 0.003), total cholesterol (*P* = 0.002), IQR/M_CAP_ (*P*<0.001), fibrosis stage (*P* = 0.001), activity grade (*P*<0.001), and steatosis grade (*P*<0.001). In subsequent multiple linear regression analysis, CAP value was independently associated with BMI (ρ = 0.214, *P* = 0.001) and IQR/M_CAP_ (ρ = −0.216, *P* = 0.001) along with histological steatosis grade (ρ = 0.455, *P*<0.001). (**Table S2 in File S1**).

### Discordance between LB and CAP

Discordance between LB and CAP was observed in 13 (8.1%) patients, whereas steatosis was underestimated on CAP in 5 (38.5%) patients and overestimated in 8 (61.5%) (**Table 2**). When patients with and without discordance between LB and CAP were compared, only the proportions of steatosis grade 3 and CAP values were significantly higher in patients with discordance (all *P*<0.05) (**Table 3**). Other clinical variables including distribution of etiologies for CLD did not significantly differ between two groups (**Table 3**).

**Table 2:** Distribution of steatosis according to liver biopsy and CAP.

Steatosis according to liver biopsy	Steatosis according to calculated cutoff CAP value			
	S0 (<250 dB/m, n = 75)	S1 (≥250 dB/m, n = 37)	S2 (≥301 dB/m, n = 19)	S3 (≥325 dB/m, n = 30)
S0 (n = 42)	39	3	**0**	**0**
S1 (n = 80)	32	31	9	**8**
S2 (n = 33)	**3**	2	9	19
S3 (n = 6)	**1**	**1**	1	3

CAP, controlled attenuation parameter.

Bold cells indicate the number of patients with discordance between LB and CAP values.

**Table 3:** Comparison of patients with and without discordance.

Variables	Patients without discordance(n = 148, 91.9%)	Patients with discordance(n = 13, 8.1%)	*P* value
**Demographic variables**			
Age (years)	49 (18–81)	52 (35–70)	NS
Male gender	95 (64.2)	7 (53.8)	NS
Body mass index (kg/m^2^)	24.4 (14.3–34.3)	24.5 (21.6–29.8)	NS
Diabetes mellitus	23 (15.5)	5 (38.5)	NS
Etiology, NAFLD/HBV/HCV/Others	64 (43.9)/45 (30.4)/26 (17.6)/12 (8.1)	7 (53.8)/4 (30.8)/2 (15.4)/0 (0.0)	NS
**Biochemical parameters**			
Aspartate aminotransferase (IU/L)	46.1±37.1	34.5±21.2	NS
Alanine aminotransferase (IU/L)	50.9±45.0	36.6±25.8	NS
Serum albumin (g/dL)	4.1±0.5	4.2±0.4	NS
Fasting glucose (mg/mL)	108.2±34.6	124.0±39.5	NS
Total cholesterol (mg/mL)	171.5±40.9	180.1±36.0	NS
Triglycerides (mg/mL)	126.2±60.4	153.7±54.9	NS
**Liver biopsy**			
Fibrosis stage			
F0–2/F3–4	99 (66.9)/49 (33.1)	10 (76.9)/3 (23.1)	NS
Activity grade			
A0–2/A3	114 (77.0)/34 (23.0)	9 (69.2)/4 (30.8)	NS
Steatosis			
S0–2/S3	144 (97.3 )/4 (2.7)	11 (84.6)/2 (15.4)	0.021
Biopsy length (cm)	18.2 (16.4–24.2)	18.6 (16.1–23.5)	NS
**Liver stiffness measurement**			
Liver stiffness value (kPa)	8.2 (2.9–75.0)	7.4 (3.0–17.5)	NS
Interquartile range (kPa)	1.0 (0.1–14.0)	1.5 (0.4–2.6)	NS
IQR/M	0.12 (0.01–0.35)	0.15 (0.06–0.26)	NS
**Controlled attenuation parameter**			
Controlled attenuation parameter value (dB/m)	250 (149–400)	327 (230–345)	0.010
Interquartile range (dB/m)	29.0 (6–72)	30.0 (15–76)	NS
IQR/M_CAP_	0.12 (0.02–0.28)	0.10 (0.05–0.33)	NS

Variables are expressed as median (range) or n (%).

NS, not significant; NAFLD, non-alcoholic fatty liver disease; HBV, hepatitis B; HCV, hepatitis C; kPa, kilopascal; IQR/M, interquartile range/median liver stiffness value; CAP, controlled attenuation parameter; IQR/M_CAP_, interquartile range/median of CAP value.

### Predictors of Discordance

**Figure 2:**
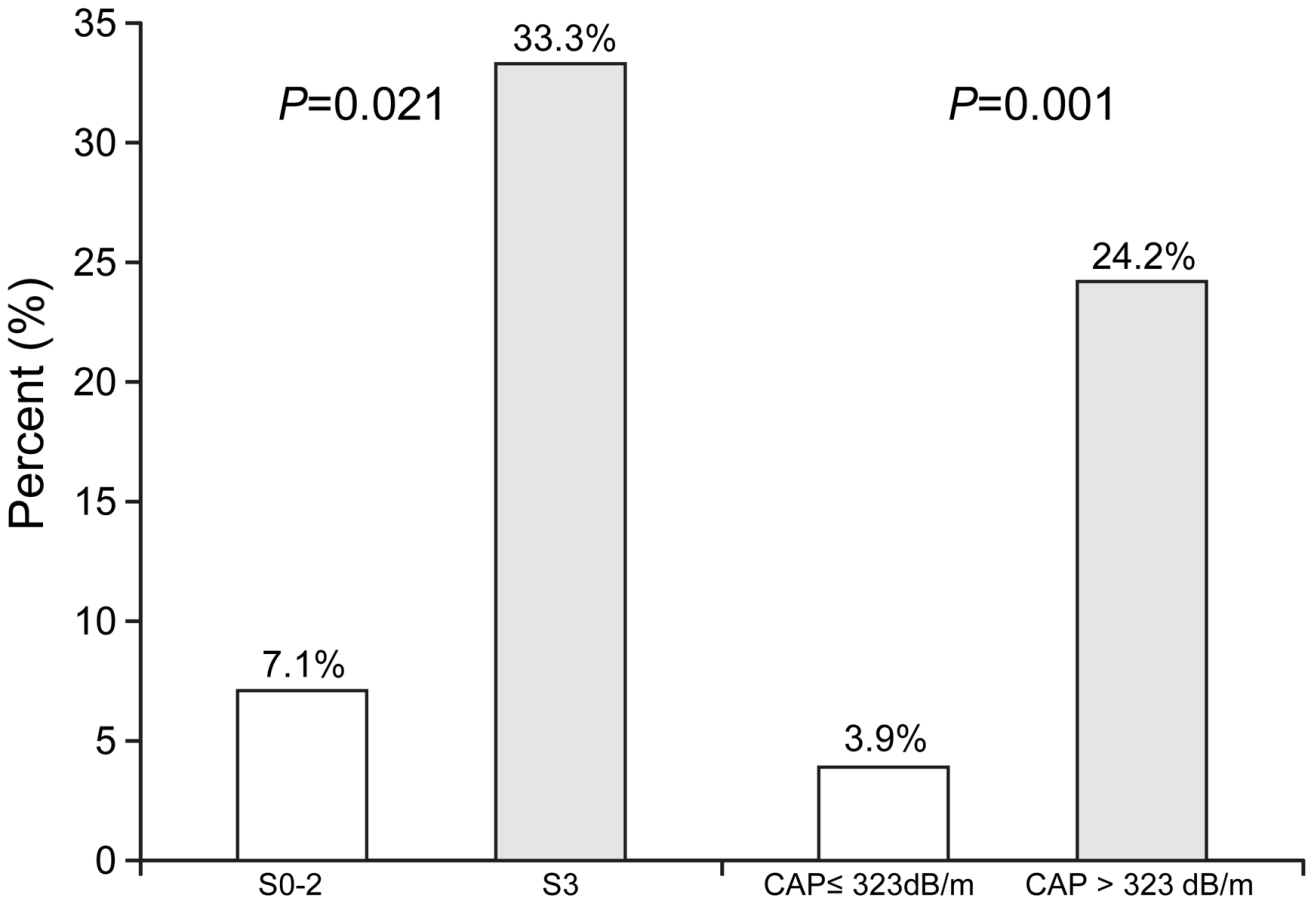
Percentage of patients with discordance according to histological steatosis grade and CAP value. The percentages of discordance between liver biopsy and CAP were significantly higher in patients with steatosis grade 3 than in those with steatosis grade 0–2 (2 of 6 [33.3%] vs. 11 of 155 [7.1%], *P* = 0.021). Additionally, patients with CAP>323 dB/m had a higher percentage of discordance than those with CAP≤323 dB/m. (8 of 33 [23.1%] vs.5 of 128 [4.9%], *P* = 0.001). CAP, controlled attenuation parameter.

In the first place, to identify independent risk factors for discordance, we performed multivariate analysis using histological parameters; the steatosis grade was entered into a multivariate analysis along with other two variables (BMI and IQR/M_CAP_) which showed an independent correlation with CAP value on linear regression analysis. The histological activity grade was entered simultaneously as a covariate into multivariate analysis to adjust for the well-known overestimating influence of TE, given that CAP is measured on the basis of TE. [22] Finally, on multivariate analysis, only steatosis grade 3 was significantly related to discordance (*P* = 0.033; OR, 9.573; 95% CI, 1.207–75.931) (**Table 4**). The percentages of discordance were significantly higher in patients with steatosis grade 3 than in those with steatosis grade 0–2 (2 of 6 [33.3%] *vs.* 11 of 155 [7.1%], *P* = 0.021) (**Fig. 2**).

**Table 4:** Independent predictors of discordance between liver biopsy and CAP.

Variables	*P* value	Odd ratio (95% CI)
**Model using histological parameters**		
Steatosis grade 3	0.033	9.573 (1.207–75.931)
Activity grade 3	NS	–
*Body mass index (kg/m^2^)	NS	–
*IQR/M_CAP_	NS	–
**Model using clinical parameters**		
CAP value (dB/m)	0.006	1.020 (1.006–1.034)
Alanine aminotransferase (IU/L)	NS	–
*Body mass index (kg/m^2^)	NS	–
*IQR/M_CAP_	NS	–

CAP, controlled attenuation parameters; CI, confidence interval; NS, not significant; IQR/M_CAP_, interquartile range/median of CAP value.

*Body mass index and IQR/M_CAP_ were incorporated into multivariate analysis due to their significant correlations with CAP value in multivariate linear regression analysis.

Similarly, we performed multivariate analysis using clinical parameters; CAP value was entered into multivariate analysis along with two other variables (BMI and IQR/M_CAP_) which were independently correlated with CAP value in linear regression analysis. The ALT level, instead of the histological activity grade, was entered simultaneously as a covariate into multivariate analysis to adjust for the well-known overestimating influence of TE, since CAP is measured on the basis of TE. [28], [29] Finally, on multivariate analysis, only CAP value was the only independent predictor of discordance (*P* = 0.006; OR, 1.020; 95% CI, 1.006–1.034). The most discriminative CAP cutoff value to predict discordance by maximizing the Youden index was 323 dB/m. Patients with CAP>323 dB/m had a higher percentage of discordance than those with CAP≤323 dB/m. (8 of 33 [24.2%] vs. 5 of 128 [3.9%], *P* = 0.001) (**Fig. 2**).

## Discussion

Although CAP showed promising results for non-invasive diagnosis of the significant steatosis, it is not obvious whether quantification of steatosis assessed by CAP could stratify severity of steatosis accurately in patients with severe steatosis. [15]–[18] Myer *et al.* reported that the diagnostic performance of CAP to identify severe steatosis was sub-optimal [16], and the ability to differentiate between steatosis grade 2 and 3 was not satisfactory in the studies by Sasso *et al.* and Ledinghen *et al.* [15], [17] Consistent with these results, a high steatotic burden (steatosis grade 3 or high CAP values) was selected as the independent risk factor of discordant results between LB and CAP in our study.

To our knowledge, this is the first study to identify the factors that influence the accuracy of CAP by using the end point of discordance between LB and CAP values. Generally, it has been known that LS values become more reliable when advanced fibrosis or cirrhosis exists and that TE can diagnose liver cirrhosis with higher accuracy. [21], [30] In contrast, CAP was more accurate in assessing less severe hepatic steatosis (≤S2) in our study as well as in those by Sasso *et al.* and Ledinghen *et al.*, although LS and CAP values were simultaneously measured from the same device. [15], [17] The reason for this opposite phenomenon of LS and CAP is unclear. However, it can be hypothesized that the correlation between ultrasonic attenuation and the amount of hepatic steatosis may be diminished, especially when the steatosis is severe. Indeed, steatosis grade 3 was selected as the only influencing factor in discordance between LB and CAP. In addition, when only clinical factors including CAP values were adjusted, only CAP values independently influenced the discordance and its most discriminative cutoff was similar to the cutoff value for diagnosing steatosis grade 3 (323 vs. 325 dB/m). These results suggest that high CAP values can be used as the single most significant factor in determining the accuracy of CAP and thus, careful diagnosis considering clinical correlations may be required when a patient shows unexpectedly high CAP values. However, since a small sample size of S3 with potential spectrum bias and interpretational variability in grading histological hepatic steatosis may have lowered the diagnostic performance of CAP in patients with high steatotic burden, [31] further studies with a well-balanced distribution of hepatic steatosis stages using objective assessment tools such as computerized morphometry are required to clarify the accuracy of CAP. [14], [31].

A high ALT levels has been identified as one of the most important confounders of LS values. [21] However, in the present study, there was no significant correlation between ALT level and CAP values, and ALT level was similar in patients with and without discordance (mean 50.9 vs. 36.6 IU/L; *P*>0.05). Similarly, histological necroinflammatory activity did not significantly influence discordance in our study, although previous studies reported overestimation due to necroinflammation. [22] Although there is still a chance that the influence of the high ALT level may have been masked due to the relatively low mean ALT level (49.8 IU/L) in our study population, our data suggest that the influence of a high ALT level or necroinflammation on CAP values seems negligible.

In our study, the effects of other clinical parameters on discordance were also investigated. First, the influence of IQR/M_CAP_ on the accuracy of CAP was explored and assessed for use as a surrogate marker for so called “reliable” criteria for CAP value as IQR/M is currently used to determine the reliability of LS value. [19] However, IQR/M_CAP_ was not selected as an independent predictor of discordance. In addition, we also tested the influence of IQR/M on discordance, but it is not associated with the diagnostic accuracy of CAP. Thus, it should be further investigated whether IQR/M_CAP_ or IQR/M can be incorporated into “reliable” criteria for CAP, although IQR/M can influence the accuracy of LS value. [19] Second, the influence of histological necroinflammatory activity and ALT level were also tested, but neither was associated with discordance, although previous studies revealed that a higher activity grade or high ALT level can cause significant overestimation of LS value. [22], [32], [33] These findings suggest that CAP has different characteristics from LS measurement and despite simultaneous measurement, should be interpreted independently in terms of the accuracy of CAP and the influence of necroinflammatory activity and ALT level. Lastly, although we hypothesized that the BMI can be a surrogate marker to predict the accuracy of CAP values considering a significant association between BMI and CAP in our cohort, the BMI was not correlated with the discordance. However, because the overall BMI of our study population was not relatively high, the potential influence of high BMI might have been masked. Thus, our results did not clarify whether adjusting for BMI would increase the accuracy of CAP interpretation; this should be investigated in future.

Our study had several limitations. First, our cohort included patients with CLDs due to various etiologies. As the diagnostic performance could vary according to the etiology, the results may have been influenced. However, other clinical variables including steatosis grade and fibrosis stage did not significantly differ according to etiologies. Additionally, recent studies demonstrated that the accuracy of CAP was similar among different etiologies including viral hepatitis and NAFLD, suggesting that heterogeneous etiologies may not have a major influence on our results. [34] Second, the small sample size of severe steatosis and relatively low mean BMI might also have led to a bias, which could be related to the low diagnostic accuracy of CAP values for diagnosis of S3 steatosis. Furthermore, the AUROC can vary according to the prevalence of each steatosis grade. Thus, our results should be confirmed in future studies with a good balance of sample size in each steatosis grade and even BMI distribution. Third, the relationship between metabolic syndrome and CAP could not be fully evaluated in this study, as the assessment of metabolic syndrome was feasible only in 60 of 161 (37.2%) patients. Although, sub-group analysis revealed that metabolic syndrome did not influence the risk of discordance between LB and CAP (data not shown, *P*>0.05), further large scale study is required to investigate the potential influence of metabolic syndrome on CAP. Lastly, we defined our end point as “discordance of two steatosis stages between LB and CAP”. Although these analytical methods have been used in several previous cross-sectional studies, [19], [22] this definition still seems to be obscure.

In conclusion, histological steatosis grade 3 and high CAP values were identified as significant factors to decrease the diagnostic performance of CAP. Thus, our results suggest that high CAP values should be interpreted carefully and that additional complementary diagnostic modality such as ultrasonography or serological steatosis prediction indices, should be used to avoid errors in this area.

## Supporting Information

File S1 **Supporting file including Tables S1 and S2. Table S1.** Liver histology and corresponding CAP values. **Table S2.** Clinicopathological variables associated with CAP.
(DOCX)
