# Correction: Factors Affecting the Accuracy of Controlled Attenuation Parameter (CAP) in Assessing Hepatic Steatosis in Patients with Chronic Liver Disease

**DOI:** 10.1371/journal.pone.0107331

**Published:** 2014-08-27

**Authors:** The PLOS ONE Staff

The fifth author’s name is misspelled. The correct name is Kyeong Hyeon Chun. The correct citation is:

Jung KS, Kim BK, Kim SU, Chon YE, Chun KH, et al. (2014) Factors Affecting the Accuracy of Controlled Attenuation Parameter (CAP) in Assessing Hepatic Steatosis in Patients with Chronic Liver Disease. PLoS ONE 9(6): e98689. doi:10.1371/journal.pone.0098689
