# Supplementary material for: Factors Affecting the Accuracy of Controlled Attenuation Parameter (CAP) in Assessing Hepatic Steatosis in Patients with Chronic Liver Disease
Source: PLoS One. 2014 Jun 5;9(6):e98689. doi: 10.1371/journal.pone.0098689 (PMC4046989; doi:10.1371/journal.pone.0098689)
Supplement: File S1 — Supporting file including Tables S1 and S2. Table S1. Liver histology and corresponding CAP values. Table S2. Clinicopathological variables associated with CAP. (DOCX) [file pone.0098689.s001.docx]

|  | | | | | | | | |  |  |  |
| --- | --- | --- | --- | --- | --- | --- | --- | --- | --- | --- | --- |
| **Table S1. Liver histology and corresponding CAP values** | | | | | | | | | | | |
| Steatosis | | CAP value (dB/m) |  | Activity grade | | CAP value (dB/m) |  | Fibrosis stage | | | CAP value (dB/m) |
| Grade | n (%) |  |  | Grade | n |  |  | Stage | | n |  |
| 0 | 42 (26.1) | 217 (149-288) |  | 0 | 1 | 155 |  | 0-1 | | 4 | 223 (155-245) |
|  |  |  |  | 1 | 2 | 213 (196-231) |  | 2 | | 18 | 215 (165-244) |
|  |  |  |  | 2 | 23 | 214 (149-288) |  | 3 | | 8 | 199 (149-250) |
|  |  |  |  | 3 | 16 | 224 (170-287) |  | 4 | | 12 | 229 (154-288) |
| 1 | 80 (49.7) | 258 (150–345) |  | 0 | 18 | 261 (173-338) |  | 0-1 | | 37 | 266 (173-345) |
|  |  |  |  | 1 | 8 | 301 (220-343) |  | 2 | | 17 | 242 (168-327) |
|  |  |  |  | 2 | 37 | 261 (150-345) |  | 3 | | 11 | 271 (150-327) |
|  |  |  |  | 3 | 17 | 242 (168-327) |  | 4 | | 15 | 261 (196-313) |
| 2 | 33 (20.5) | 331 (234–400) |  | 0 | 4 | 357 (336-380) |  | 0-1 | | 19 | 336 (249-383) |
|  |  |  |  | 1 | 12 | 331 (249-375) |  | 2 | | 8 | 316 (258-360) |
|  |  |  |  | 2 | 13 | 331 (258-400) |  | 3 | | 3 | 244 (234-313) |
|  |  |  |  | 3 | 4 | 256 (234-303) |  | 4 | | 3 | 379 (318-400) |
| 3 | 6 (3.7) | 326 (230–347) |  | 0 | 0 | - |  | 0-1 | | 5 | 333 (230-347) |
|  |  |  |  | 1 | 5 | 323 (230-347) |  | 2 | | 1 | 283 |
|  |  |  |  | 2 | 1 | 330 |  | 3 | | 0 | - |
|  |  |  |  | 3 | 0 | - |  | 4 | | 0 | - |
| CAP, controlled attenuation parameter. CAP values were described as median (range).  CAP values were described as median (range). | | | | | | | | | | | |

| **Table S2.** Clinicopathological variables associated with CAP | | | | |
| --- | --- | --- | --- | --- |
| Variable | Univariate |  | Multivariate | |
|  | *P* value |  | ρ | *P* value |
| Body mass index (kg/m^2^) | <0.001 |  | 0.214 | 0.001 |
| Alanine aminotransferase (IU/L) | 0.003 |  | - | NS |
| Total cholesterol (mg/mL) | 0.002 |  | - | NS |
| IQR/M_CAP_ | <0.001 |  | -0.216 | 0.001 |
| Fibrosis stage | 0.001 |  | - | NS |
| Activity grade | <0.001 |  | - | NS |
| Steatosis grade | <0.001 |  | 0.455 | <0.001 |
| CAP, Controlled attenuation parameters; NS, not significant; IQR/M_CAP_, interquartile range/median of CAP value. | | | | |
